# Artificial intelligence for diabetic retinopathy in low-income and middle-income countries: a scoping review

**DOI:** 10.1136/bmjdrc-2023-003424

**Published:** 2023-08-02

**Authors:** Charles R Cleland, Justus Rwiza, Jennifer R Evans, Iris Gordon, David MacLeod, Matthew J Burton, Covadonga Bascaran

**Affiliations:** 1 International Centre for Eye Health, Faculty of Infectious and Tropical Diseases, London School of Hygiene & Tropical Medicine, London, UK; 2 Eye Department, Kilimanjaro Christian Medical Centre, Moshi, United Republic of Tanzania; 3 Tropical Epidemiology Group, Department of Infectious Disease Epidemiology, London School of Hygiene & Tropical Medicine, London, UK; 4 National Institute for Health Research Biomedical Research Centre for Ophthalmology, Moorfields Eye Hospital NHS Foundation Trust and UCL Institute of Ophthalmology, London, UK

**Keywords:** diabetic retinopathy, information technology, public health, developing countries

## Abstract

Diabetic retinopathy (DR) is a leading cause of blindness globally. There is growing evidence to support the use of artificial intelligence (AI) in diabetic eye care, particularly for screening populations at risk of sight loss from DR in low-income and middle-income countries (LMICs) where resources are most stretched. However, implementation into clinical practice remains limited. We conducted a scoping review to identify what AI tools have been used for DR in LMICs and to report their performance and relevant characteristics. 81 articles were included. The reported sensitivities and specificities were generally high providing evidence to support use in clinical practice. However, the majority of studies focused on sensitivity and specificity only and there was limited information on cost, regulatory approvals and whether the use of AI improved health outcomes. Further research that goes beyond reporting sensitivities and specificities is needed prior to wider implementation.

## Introduction

The application of artificial intelligence (AI) is anticipated to have a considerable impact in many areas of our lives over the coming decades, not least in healthcare.^1^ However, implementation of AI into clinical practice remains limited.^2^

Ophthalmology is a leading specialty in the development of healthcare AI.^3^ In 2018, the first autonomous AI-based medical device to obtain approval from the Food and Drug Administration (FDA) in the USA was the IDx-DR system for the detection and grading of diabetic retinopathy (DR) from retinal photographs.^4^ Ophthalmology is a potential exemplar specialty in the application of medical AI, with its use in the context of DR leading the way.^3^

DR is a common complication of diabetes and is a leading cause of blindness globally.^5^ DR is often asymptomatic until at an advanced stage, when it is less amenable to treatment; therefore, screening is recommended to prevent sight loss. The early detection and treatment of people with sight-threatening DR substantially reduces the risk of severe visual loss in persons with this stage of disease.^6^

However, many low-income and middle-income countries (LMICs) have no or very limited screening services for DR. As the projected increases in the number of people with diabetes, and consequently DR, will disproportionally affect LMICs,^7^ this is a concern. Unless improved screening and treatment services are developed and implemented in LMICs, preventable sight loss from DR will inevitably rise.

A major barrier to the implementation of diabetic eye care services in LMICs is a lack of trained staff. For example, in sub-Saharan Africa (SSA), the region projected to see the largest proportionate increase in the number of people living with DR (up by 143% to 16.3 million people by 2045),^8^ there are 2.5 ophthalmologists per million population against a global average of 37.5.^9^ Healthcare AI, which task shifts away from clinical staff, has arguably a greater potential to improve clinical care in LMICs, where human resources for healthcare are most stretched.

The aim of this scoping review is to provide eye care staff, policy makers and researchers with an overview of the literature relating to the use of AI for DR in LMICs to guide clinical trials and the potential implementation of AI tools for DR into clinical pathways.

### Research questions

Our research questions are:

1. What AI systems have been used for DR either in, or on data from, populations from LMICs?
2. What are the performance metrics and characteristics of the AI tools used?

Performance metrics include diagnostic accuracy and implementation outcomes (acceptability, fidelity, etc); characteristics include regulatory approvals, technical specifications, cost information and data management functionality.

The research questions are broad to provide a comprehensive overview of the literature, beyond diagnostic accuracy, in order to guide the use of AI for DR in clinical pathways in LMICs.

## Methods

The study is reported according to the Preferred Reporting Items for Systematic reviews and Meta-analyses extensions for Scoping Reviews guidelines.^10^ The protocol was registered on the Open Science Framework repository.^11^

A scoping review was considered the most appropriate methodology for answering the research questions. Our methodological approach was informed by the published guidelines for conducting scoping reviews.^10 12^ The core search concepts for the scoping review were DR, AI and LMICs.

### Search strategy and selection criteria

We searched MEDLINE (Ovid), Embase (Ovid), Global Health (Ovid) and the Cochrane Central Register of Controlled Trials on the Cochrane Library on November 29, 2022. A Cochrane Eyes and Vision Information Specialist (IG) developed the search strategies. The searches were constructed using Medical Subject Headings and free-text terms for the following topic areas: “artificial intelligence”, “diabetic retinopathy” and “low- and middle-income countries”. No language limits were applied to the searches. The searches were limited to 2008 onwards. In view of the substantial advances in technology since this date, any publications prior to 2008 are unlikely to be relevant to our objectives. The search strategies are presented in online supplemental appendix 1.

10.1136/bmjdrc-2023-003424.supp1 Supplementary data

In order to capture studies using imaging data from LMICs, we additionally searched for publications that used 14 publicly available ophthalmic imaging datasets which are from LMICs. A list of these LMIC datasets is available in online supplemental appendix 2. This list was informed by a recent review of all publicly available ophthalmic datasets in *Lancet Digital Health*, which detailed the country of origin of the imaging data.^13^

10.1136/bmjdrc-2023-003424.supp2 Supplementary data

The inclusion and exclusion criteria were defined before conducting the review but, in keeping with guidelines for scoping reviews,^10^ articles were selected during the title and abstract screening if (1) they referred to the use of AI in the context of DR and (2) were conducted in, or using data from people living in an LMIC. All primary research studies were included.

Reviews were excluded but their reference lists were searched for any primary articles that were not included from our original search. Gray literature and conference abstracts were excluded as they do not provide sufficient evidence to inform clinical trials or practice.

AI was defined as any technology, computer software or algorithm that makes an autonomous decision in a manner that mimics human cognition.^14^ DR is a complication of diabetes and we included articles that discussed DR or diabetic macular edema (DME). LMICs were defined according to the World Bank definition for 2021.^15^

The differences between our protocol and the review included using the 2021 World Bank definition of LMIC as opposed to 2019 definition stated in our protocol and the addition of a google search to identify additional relevant information about the identified AI systems.

### Selection of studies

All identified records were imported into Covidence (Covidence systematic review software, Veritas Health Innovation, Melbourne, Australia, available at www.covidence.org) for screening. Two authors (CRC and CB) independently reviewed each title and abstract and excluded those not meeting the inclusion criteria. Disagreements were resolved by discussion and consensus. The full texts were then again reviewed independently by two reviewers (CRC and CB) to determine which articles should be included in the data extraction phase, with all disagreements resolved by discussion and consensus.

### Data charting process

A data extraction form was developed in Covidence based on the scoping review questions and was piloted by two reviewers (CRC and CB). The form was refined based on discussion and finalized prior to extraction. Data extraction was then carried out for each publication independently by two reviewers (CRC and JR). After extraction, all differences were resolved by discussion and consensus.

### Data items

Characteristics of publication:

- Title, year of publication, journal
- Affiliation of first author
- Sources of funding
- Stated conflicts of interests for any authors

Characteristics of the AI tool:

- Stated name of AI tool
- Function/Intended use of AI tool
- Would the published study be considered an external validation
- Regulatory approvals
- Purchase cost of AI software
- DR classification system used

External validation was defined as the testing of AI on a new set of data entirely separate from the training dataset.^16^ This is a crucial step in the development of AI as it demonstrates that an AI model can work in patients and populations external to the development population.

A Google search of the identified AI systems was performed to extract information on the regulatory approvals and purchase cost of the AI not accessible from the publications.

Characteristics of data used to assess performance of AI:

- Type of imaging data used (retinal photographs, optical coherence tomography (OCT))
- Country of origin of data
- Data collected retrospectively or prospectively
- Details of reference standard and arbitration process

Reported performance of AI tool:

- Sensitivity, specificity, area under the curve (AUC)

Implementation-related outcomes:

- Economic evaluation outcomes
- Implementation research outcomes (fidelity, acceptability, adoption, sustainability)
- Any other reported outcome data not already captured

### Synthesis of the results

We conducted a descriptive analysis of the study characteristics, study methods and of the AI tools. Study characteristics included the location of the study (defined as where the imaging data was from), year of publication, funding and conflicts of interest and first author affiliation. The study methods captured whether the data used to train/test the AI were collected prospectively or retrospectively, how many images or participants were included in the dataset, who provided the reference standard and details of the arbitration process. For the AI tool, we captured what task the AI was designed to perform, the performance of the AI for its given task, the name and/or developer if stated, regulatory or cost data and any implementation research outcomes.

The location of all included studies was displayed visually on a map. The studies were then coded into those that were, and were not, considered external validations; key data of studies that were considered external validations were summarized and tabulated. Externally validated studies were then coded into those that had a named AI or a stated developer and those that did not. For those with a named AI and/or stated developer, the sensitivity, specificity and AUC of the AI tool in detecting referable DR was tabulated along with the reference standard, arbitration process and any other key outcome measures.

A consultation stage, which is considered optional in scoping reviews, was not undertaken.^17^

## Results

**Figure 1:**
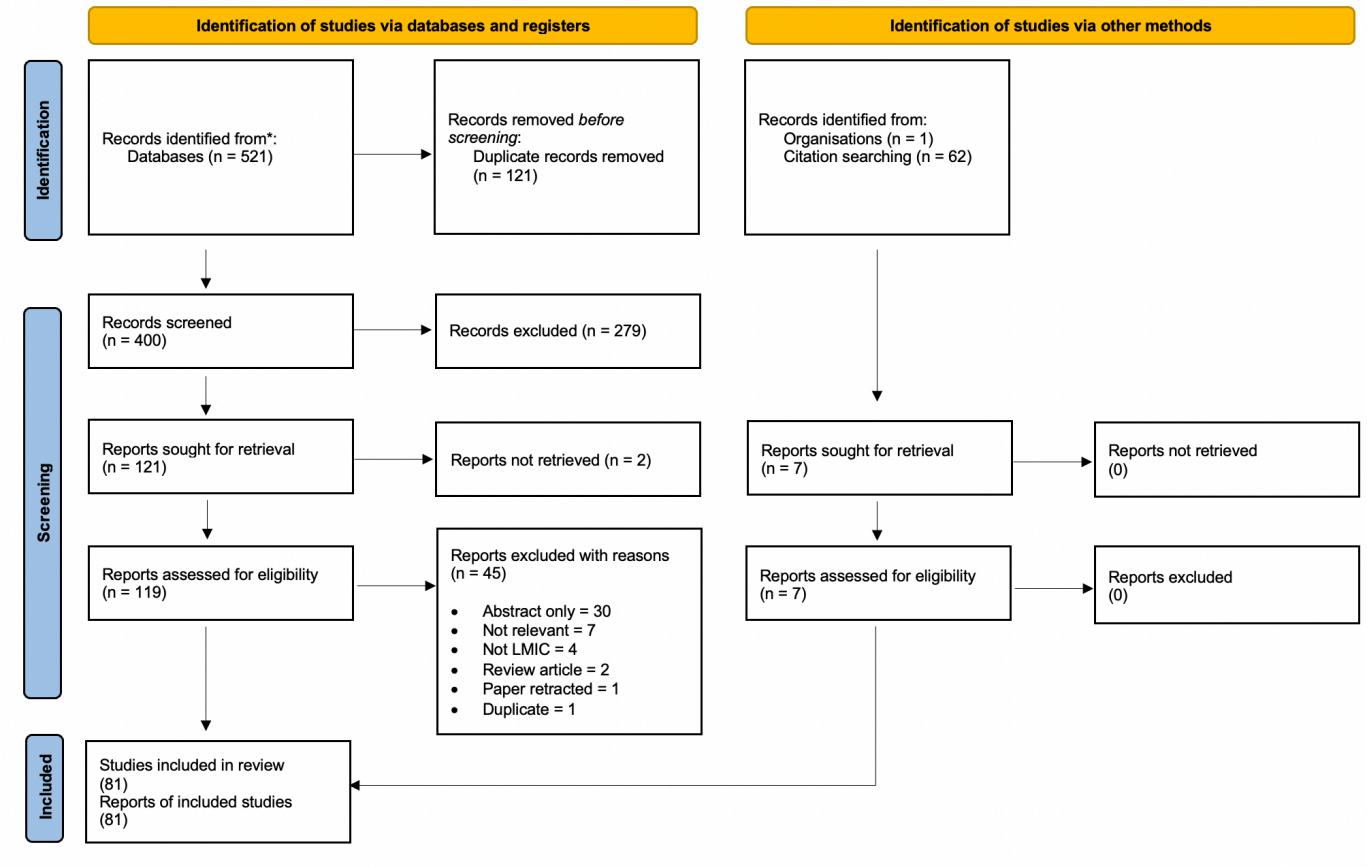
Preferred Reporting Items for Systematic Reviews and Meta-Analyses (PRISMA) 2020 flow diagram. LMIC, low-income and middle-income country.

Figure 1 shows a Preferred Reporting Items for Systematic Reviews and Meta-Analyses flow chart outlining the selection process for the included articles.

The searches last run on November 29, 2022 retrieved a total of 521 records. After 121 duplicate records were removed, 400 unique records were screened by title and abstract. A total of 279 records were excluded at the title and abstract stage and 121 records went forward to full-text review. Two reports could not be sourced, therefore a total of 119 reports of studies were assessed for potential inclusion in the review. After reading the full texts, 74 studies were included and 45 studies were excluded.

The reference lists of 62 reviews were assessed and an additional 7 studies not identified in our primary searches were included. Therefore, a total of 81 studies met our inclusion criteria and were included in the analysis.

### Characteristics of the publications

**Figure 2:**
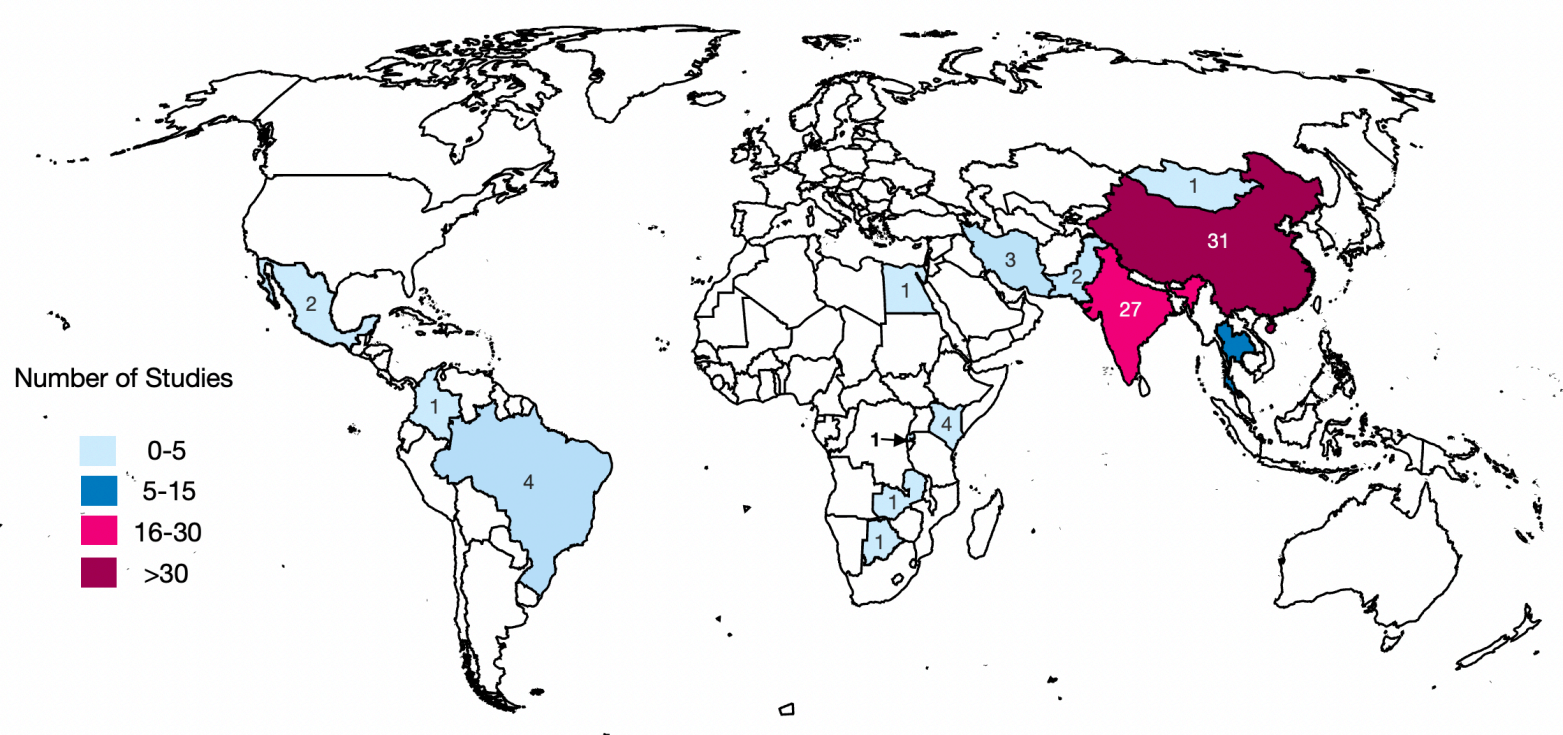
World map showing the distribution of artificial intelligence research for diabetic retinopathy in low-income and middle-income countries. The total number of studies exceeds 81; this is because some studies used data from more than one country.

The majority of the identified studies were undertaken in three countries: India, China and Thailand (figure 2) with 65% (n=53) of publications in 2020, 2021 and 2022. The majority of first author affiliations were from institutions in LMICs (n=62; 77%); however, of the studies conducted in SSA (n=7; 9%), only one first author was affiliated with an institution in an African country.^18^

The primary aim of most studies (n=72; 89%) was to assess performance of the AI for a specific task. These studies either focused on a description of AI tool development and performance in the training dataset (n=29; 36%) or a description of performance in an external dataset (n=43; 53%) that was entirely separate from the training data; therefore, meeting our definition of external validation.

### Characteristics of the AI tools

The function of the majority of the AI tools identified (n=49; 60%) was to automate retinal photograph interpretation and produce a DR grade. Of the remaining studies, five focused on using AI for automating the interpretation of OCT imaging for DME^19–23^ and one study evaluated the performance of AI in fundus fluorescein angiography interpretation.^24^ Two studies considered the performance of an AI tool for grading DR and multiple other retinal conditions^25 26^ and a further two studies considered the performance of two different AI models for grading DR, possible glaucoma and age-related macular degeneration/referable macular diseases.^27 28^

One study used an AI tool to identify macular edema from two-dimensional retinal photographs^29^; one study used an AI tool to identify, in persons with diabetes, fundus images without DR (ie, normal fundi)^30^; one study used heat maps to aid with the ‘black box’ phenomenon in an attempt to understand why an AI model might produce false positives in the context of DR grading^31^ and one study used AI to inform the photographer whether images taken with a handheld smartphone without mydriasis were gradable for DR and to assess if this could reduce the number of ungradable images captured.^32^

Seven studies focused on identifying specific features on a retinal image, such as the presence of hard exudates or vessel bifurcation, which are informative when grading an image for DR.^33–39^

Two studies reported the use of an AI tool to predict the likelihood of DR progression^40 41^; one study assessed whether AI-assisted image grading can improve human grading^42^; two studies assessed the impact of using an AI model on patient flow within DR screening services^43 44^ and one study used an AI model to collect DR prevalence data.^45^

Two studies had an implementation research focus^46 47^ and two studies evaluated the cost-effectiveness of using AI for DR screening.^48 49^

### Externally validated studies

A total of 43 studies (52%) met our definition of external validation. In summary, the majority (n=33; 77%) reported the use of AI for automating the interpretation of retinal images and producing a DR grade and 25 (58%) used prospectively collected data.

Thirty-one (72%) of the externally validated studies had a named AI tool and/or details of the company that developed the tool; therefore, 12 studies did not state the AI name or details of the developer. Of those studies with a named AI and/or developer details, 16 had direct declared conflicts of interests relating to either the AI software or the company that developed the AI and 9 of those studies were funded by the company that developed the AI used in the respective study.

Only four studies stated that the AI tool assessed was commercially available^19 50–52^; however, the company referred to in two of these publications (Visulytix) has ceased trading.

### AI model performance

**Figure 3:**
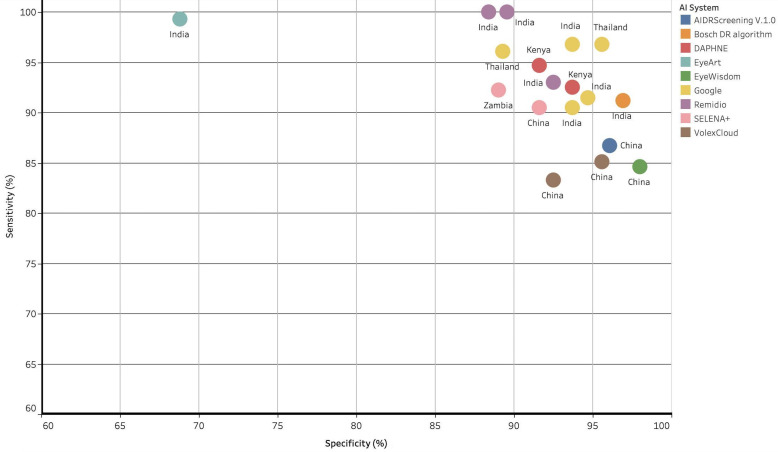
Scatter plot showing sensitivity and specificity for the detection of referable diabetic retinopathy (DR) for artificial intelligence (AI) systems with a stated name and/or developer. Data points are color coded by AI system and are labeled with the country where either the study was undertaken or where the data used in the study are from. Only those AI systems with a stated name and/or developer and which reported sensitivity and specificity for detecting referable DR are displayed.

As noted above, the majority of studies (n=50; 61%) reported the use of an AI tool for automated retinal image interpretation in order to produce a DR grade. Table 1 details the performance of the externally validated AI models which also had a stated name and/or developer for the detection of referable DR. The sensitivities and specificities for the detection of referable DR in these studies ranged from 83.3%–100% to 68·8%–98%, respectively (figure 3). One study reported that AI performance was not significantly affected by gender.^53^

**Table 1:** Reported sensitivity, specificity and AUC for the detection of referable DR for externally validated studies that state either the name of the AI tool or the developer

Study	Country	AI system name/developer	Study type	Number of participants or images	Who provided the reference standard	DR classification system used	Sensitivity (95% CI) for referable DR	Specificity (95% CI) for referable DR	AUC (95% CI) for referable DR	Referable DR threshold	Other reported outcomes
Sosale *et al*^73^	India	Medios AI	Prospective	922 participants	Five retinal specialists	ICDRS	93% (91.3 to 94.7)	92.5% (90.8 to 94.2)	0.88	Moderate NPDR or worse and/or presence of DME	None
Bahl and Rao^74^	India	Medios AI	Prospective	250 participants	None	NS	NS	NS	NS	NS	197 gradable images obtained from 250 participants
Natarajan *et al*^75^	India	Medios AI	Prospective	231 participants	Two ophthalmologists	ICDRS	100%	88.4%	NS	Moderate NPDR or worse and/or presence of DME	None
Jain *et al*^51^	India	Medios AI	Prospective	1378 participants	Two vitreo-retinal surgeons	ICDRS	100% (94.72 to 100)	89.55% (87.76 to 91.16)	NS	Moderate NPDR or worse (macular signs not specified)	None
Ting *et al*^67^	China, Australia, Singapore, USA	SELENA+	Retrospective	18 913 participants	Ophthalmologist and retina specialist	ICDRS	NS	NS	0.963 (0.956 to 0.969)	Moderate NPDR or worse (macular signs not specified)	DLS system was quicker at performing grading than humans
Bellemo *et al*^53^	Zambia	SELENA+	Retrospective	1574 participants	Two trained graders	ICDRS	92.25% (90.10 to 94.12)	89.04% (87.85 to 90.28)	0.973 (0.969 to 0.978)	Moderate NOPDR or worse, DME or an ungradable image	None
Ting *et al*^27^	China, Mexico, Singapore	SELENA+	Retrospective	14 880 participants (in validation set)	Varied between datasets	ICDRS	90.5% (87.3 to 93.0)	91.6% (91.0 to 92.2)	0.936 (0.925 to 0.943)	Moderate NODR or worse, DME or an ungradable image	DLS performance for detecting possible glaucoma and AMD also reported
Sayres *et al*^76^	India and USA	Google	Retrospective	1612 participants	Three retinal specialists	ICDRS	91.5%	94.7%	NS	Moderate or worse DR (macular signs not specified)	Human graders assisted by AI improved grading accuracy
Raumviboonsuk *et al*^66^	Thailand	Google	Retrospective	7517 participants	Two retinal specialists	ICDRS	96.8% (89.3 to 99.3)	95.6% (98.3 to 98.7)	98.7% (97.7 to 99.5)	Publication did not specify RDR, these figures are taken for the detection of moderate NPDR or worse. Referable DME data were also reported	Algorithm was statistically significantly better than local Thai graders
Gulshan *et al*^77^	India	Google	Prospective	3049 participants	A trained grader and a retinal specialist	ICDRS	Aravind: 88.9% (85.8 to 91.5) Sankara: 92.1% (90.1 to 93.8)*	Aravind: 92.2% (90.3 to 93.8) Sankara: 95.2% (94.2 to 96.1).*	*Aravind: 0.963. Sankara: 0.980	Moderate NPDR or worse and/or presence of DME	None
Gulshan *et al*^78^	India and USA	Google	Retrospective	Does not specify how many images from India used to train model	Seven and eight ophthalmologists graded the images from the Messidor-2 and EyePACS-1 datasets, respectively	ICDRS	EyePACS-1: 97.5% (95.8 to 98.7) Messidor-2: 96.1% (92.4 to 93.8)†	EyePACS-1: 93.4% (92.8 to 94.0) Messidor-2: 93.9% (92.4 to 95.3)†	EyePACS-1: 0.991 (0.988 to 0.993) Messidor-2: 0.990 (0.986 to 0.995)†	Moderate NPDR or worse and/or presence of DME	Algorithm performance tested at different cut-offs (high sensitivity, high specificity. etc)
Bora *et al*^40^	Thailand and USA	Google	Retrospective	6791 participants	One ophthalmologist graded each image from a pool of 11	ICDRS	N/A	N/A	N/A	N/A	AUC for predicting risk of developing DR at 2 years from normal fundus image at baseline 0.70 (0.67 to 0.74)
Ruamviboosnuk *et al*^55^	Thailand	Google	Prospective	7651 participants	Three retinal specialists	ICDRS	95.1% (91.8 to 97.9)	89.3% (85.8 to 92.3)	NS	Moderate NPDR or worse, DME or an ungradable image	Qualitative interviews revealed that nurses positively viewed the system but concerns were expressed about images deemed ungradable by the DLS and the additional time required for image upload
Al-Turk *et al*^79^	Kenya, China, Saudi Arabia	DAPHNE	Retrospective	Kenya: 24 700 images China: 15 000 images	China: NS Kenya: Moorfields Eye Hospital retinal image reading center	Kenya: ICDRS China: UK NSC guidelines	Kenya: 94.28% (93.1 to 95.22) China: 95.51% (93.1 to 97.5)	Kenya: 92.12% (88.27 to 93.33) China: 91.11% (85.11 to 92.63)	NS	UK NSC guideline and ICDRS (moderate or worse DR) classifications used to determine referral threshold including presence of DME	None
Al-Turk *et al*^80^	Kenya, China, Saudi Arabia	DAPHNE	Retrospective	49 726 images	NS	Kenya: ICDRS China: UK NSC guidelines	Kenya: 92.03% (91.2 to 92.9) China: 92.9% (91.4 to 94.2)	Kenya: 93.0% (92.7 to 93.4) China: 94.4% (94.1 to 97.8)	NS	Kenya: moderate NPDR or worse using ICDRS criteria and/or presence of DME China: UK NSC cut-off used (includes presence of DME)	Ability of DAPHNE to detect non-DR pathology was assessed; sensitivity and specificity data not reported due to small number of cases
Rajalakshmi *et al*^81^	India	EyeArt software (V.2.1.0)	Prospective	301 participants	Two ophthalmologists	ICDRS	99.3% (96.1 to 99.9)	68.8% (61.5 to 76.2)	NS	Moderate NPDR or worse and/or presence of DME	None
Zhang *et al*^56^	China	VolexCloud	Prospective	15 805 participants	Two specialist graders	ICDRS	83.3% (81.9 to 84.6)	92.5% (92.1 to 92.9)	NS	Moderate NPDR or worse (DME assessed separately)	Prevalence data in Chinese population reported
Li *et al*^57^	China	VolexCloud	Prospective	11 476 participants	One certified retinal specialist	ICDRS	85.1% (83.4 to 86.6)	95.6% (94.6 to 96.6)	0.942 (0.920 to 0.964)	Moderate NPDR or worse (macular signs not specified)	389 images were ungradable using DLA but were gradable by humans
Lin *et al*^25^	China	Comprehensive AI Retinal Expert	Prospective	18 163 images in external validation dataset	Three ophthalmologists	NS	NS	NS	‡1. 0.960 (0.953 to 0.966) 2. 0.999 (0.998 to 1.000) 3. 0.918 (0.887 to 0.944)	Moderate NPDR or worse (macular signs not specified)	Performance of DLS in detecting 14 different retinal conditions reported
Ming *et al*^82^	China	EyeWisdom	Prospective	199 participants	Two ophthalmologists	ICDRS	84.6% (54.6 to 98.1)	98.0% (94.3 to 99.6)	0.913 (0.797 to 1.00)	More than mild NPDR (macular signs not specified)	None
Pei *et al*^83^	China	EyeWisdom	Prospective	549 participants	Two ophthalmologists	ICDRS	NS	NS	NS	NS	Sensitivity, specificity and AUC provided for each level of DR
Hao *et al*^58^	China	EyeWisdom	Prospective	3796 participants	Two attending physicians	ICDRS	NS	NS	NS	NS	Reported sensitivity and specificity data for the presence of DR
Bawankar *et al*^84^	India	Bosch DR algorithm	Prospective	564 participants	The investigators	American Academy guidelines	91.18%	96.9%	NS	Figures refer to presence or absence of DR (macular signs not specified)	Bosch algorithm showed favorable performance for non-mydriatic photos
Hansen *et al*^85^	Kenya	Iowa DR detection software	Retrospective	3460 participants	Moorfields Eye Hospital retinal image reading center	ICDRS	NS	NS	NS	NS	Reported figures for the detection of the presence of DR
Dong *et al*^26^	China	Retinal Artificial Intelligence Diagnosis System	Retrospective	110 784 participants	Two ophthalmologists	NS	NS	NS	0.981 (0.980 to 0.982)	NS	The AI system detects 10 retinal disorders
Yang *et al*^59^	China	AIDRScreening V.1.0	Prospective	962 participants	Two graders	Chinese Ophthalmic Society guidelines	86.72% (83.39 to 90.05)	96.06% (94.14 to 97.54)	NS	Stage II or worse using the Chinese Ophthalmic Society guidelines	None
Nunez do Rio *et al*^52^	India	VISUHEALTH-AI DR (software V.1.8)	Prospective	31 608 participants	Two optometrists or ophthalmologists	ICDRS	72.08% (70.68 to 73.46)	85.65% (85.11 to 86.18)	NS	Moderate NPDR or worse and/or presence of DME	Assessed AI system performance in non-mydriatic images
Mathenge *et al*^18^	Rwanda	Cybersight	Randomized controlled trial	823 participants	NS	NS	NS	NS	NS	NS	Proportion of persons referred after DR screening who attended follow-up reported
Li *et al*^68^	China	DR DL Network (V.201710242)	Prospective	8012 participants	Two graders in reading center	ICDRS	NS	NS	NS	UK NSC guideline used to determine referral threshold (includes presence of DME)	AI took 19.2 min to provide screening report after image capture vs 1.3 days for manual graders

*Study conducted in two sites; data presented separately for the two study sites.

†High-sensitivity cut-off data presented.

‡AUC presented from three different external testing datasets.

AI, artificial intelligence; AMD, age-related macular degeneration; AUC, area under the curve; DLA, Deep learning algorithm; DLS, deep learning system; DME, diabetic macular edema; DR, diabetic retinopathy; ICDRS, International Classification of Diabetic Retinopathy Severity; N/A, not available; NPDR, Non-proliferative diabetic retinopathy; NS, not stated; NSC, National Screening Committee; RDR, referable diabetic retinopathy.

As Visulytix, the company responsible for developing the Pegasus AI system, has ceased trading, those two studies were not included in table 1. One study relating to the DAPHNE AI system was also not included in table 1 as it identified fundus images for the presence of microaneurysms only.

The majority of studies used the International Clinical Diabetic Retinopathy Severity scale^54^ classification system with a threshold of moderate NPDR or worse defining referable DR. A clear description of who determined the reference standard DR grade along with the arbitration process was provided by the majority of studies, although the arbitration methodology differed between studies (see table 1 and online supplemental table 1).

10.1136/bmjdrc-2023-003424.supp3 Supplementary data

The majority of studies excluded images deemed ungradable by the human graders from the analyses. However, five studies did compare images deemed ungradable by human graders with the AI model gradings. These studies all showed that the AI tools considered a higher number of images as ungradable when compared with the human graders.^52 55–58^

Two of the five studies that tested the performance of an AI tool for detecting DME from OCT imaging met our definition of external validation. The Pegasus-OCT system detected age-related macular degeneration (AMD) and DME with a minimum area under the receiver operating characteristic of 99% and 98%, respectively^50^ and Tang *et al* reported an AUC of >0.906 in all the external test sets for detecting the presence of DME.^20^

### Other outcome measures

Three studies reported that the AI tool was a registered medical device in China^45 58 59^ and one study reported that the AI tool was registered as a class IIa medical device.^52^ No study stated the cost of the AI tool.

We identified two economic evaluation studies undertaken in China and Brazil.^48 49^ AI was found to be more cost-effective than the standard of care in the study undertaken in China but not in Brazil.

Our Google search of named AI systems or those with a stated developer revealed commercial websites for the Medios AI (Remidio Innovative Solutions),^60^ SELENA+ (EyRIS),^61^ EyeArt (Eyenuk),^62^ RAIDS (SightAI Technology)^63^ and EyeWisdom (Visionary Intelligence (Vistel))^64^ systems. Cybersight is provided as a free (non-commercial) software by Orbis International.^65^ No website provided any cost information other than Cybersight/Orbis, which stated their software is free to use in LMICs.

EyeArt’s website stated their AI system has US FDA clearance, CE marking as a class IIa medical device in the European Union and a Health Canada license and the SELENA+/EyRIS website stated that their software has both a Health Science Authority certification from Singapore and is CE marked. There were no details of any regulatory approvals on the Medios AI, RAIDS, EyeWisdom or Cybersight/Orbis website.

Three studies reported implementation research outcomes. A study undertaken in Brazil discussed the feasibility of using AI for DR screening and mainly highlighted the need to raise awareness of diabetic eye disease within the population.^47^ The second study was undertaken in Thailand when Google’s AI system was implemented in an active clinical pathway.^46^ The paper reported challenges with fidelity, particularly highlighting poor internet connectivity and suboptimal lighting affecting retinal image capture as issues, as well as acceptability concerns from nursing staff involved in DR screening. A third study in Rwanda demonstrated that for persons screened for DR, AI with a point-of-care referral decision significantly increased the proportion of persons referred from screening who attended the referral eye clinic.^18^

One study used deep learning to predict the 2-year progression from no DR on retinal imaging to signs of DR. The reported AUC was 0.70 (95% CI 0.67 to 0.74) using the deep learning model alone and this increased marginally to 0.71 (95% CI 0.68 to 0.75) when additional clinical risk factors, notably hemoglobin A1c, were added to the model.^40^

Two studies assessed the performance of an AI tool for the detection of multiple retinal pathologies, including DR.^25 27^ The SELENA+ AI system can detect DR, possible glaucoma and AMD. Ting *et al* reported an AUC for the SELENA+ AI system of 0.942 (95% CI 0.929 to 0.954), sensitivity of 96.4% (95% CI 81.7% to 99.9%) and specificity of 87.2% (95% CI 86.8% to 87.5%) for possible glaucoma and an AUC of 0.931 (95% CI 0.928 to 0.935), sensitivity of 93.2% (95% CI 91.1% to 99.8%) and specificity of 88.7% (95% CI 88.3% to 89.0%) for referable AMD. The Comprehensive AI Retinal Expert system is a DLS designed to detect 14 retinal abnormalities from fundus imaging (including DR).^25^ The mean AUC for the detection of the 14 retinal pathologies in the three external test sets was 0.940 (SD 0.035), 0.965 (SD 0.031) and 0.983 (SD 0.042). This ranged from 0.861 (95% CI 0.788 to 0.922) for referable hypertensive retinopathy to 0.999 (95% CI 0.999 to 1.000) for geographic atrophy and retinitis pigmentosa; the AUC for referable DR in the non-Chinese ethnicity external dataset was 0.960 (95% CI 0.953 to 0.966).^25^

Other outcomes reported included a pragmatic comparison of Google’s AI to local Thai graders. The AI had a sensitivity of 0.968 (range: 0.893–0.993), specificity of 0.956 (range: 0.983–0.987) and an AUC of 0.987 (range: 0.977–0.995), compared with a sensitivity of 0.734 (range: 0.4071–0.914) and a specificity of 0.980 (range: 0.939–1.000) for the regional graders; this difference was statistically significant (p<0.001).^66^ Another study from the Google health team reported the performance of a DLS in predicting macular edema from two-dimensional retinal photographs with a sensitivity of 81% and a specificity of 80%.^29^ Some studies reported on the efficiency gains achieved when using AI-supported fundus image grading, highlighting the fact that patients received their screening result much more quickly when using AI compared with human graders.^67–69^

## Discussion

There is considerable potential for AI to improve health services, particularly in LMICs. Ophthalmology is a potential exemplar medical specialty for healthcare AI, with its use in DR most advanced. We have identified 81 studies detailing the use of AI tools in LMICs in the context of DR. Over half of these report the use of AI to automate retinal image grading for DR.

Of the studies identified in this review, 43 were considered external validations. Thirty-one of those had a named AI and/or a stated developer. The reported sensitivities and specificities of these AI tools ranged from 83·3%–100% to 68·8%–98%, respectively providing evidence to support use in clinical practice. Google’s AI software, SELENA+ (EyRIS), EyeWisdom (Visionary Intelligence) and Medios AI (Remidio Innovation Solutions) accounted for about half of these publications and 13 were undertaken in, or on data from, China.

However, the majority of these 31 studies excluded ungradable images from the analyses and those that did not reported that the AI models considered a higher number of images ungradable compared with the reference standard human gradings. This suggests that if AI systems are used prospectively in active clinical pathways, when ungradable images cannot be excluded, performance is likely to be reduced. If AI tools consider a higher proportion of images as ungradable, which typically trigger a refer outcome, this could result in more false positive cases being referred to and attending ophthalmology clinics, which are already under-resourced in many LMICs.

AI tools with other functions, including predicting the risk of DR progression and the use of a single AI model to detect multiple retinal diseases, give an indication of potential future developments. However, it is less clear from the literature if and how such systems can integrate into clinical pathways and whether their use will translate into improved health outcomes.

The majority of studies identified were conducted in two countries: India and China. These two countries account for a substantial proportion of the global population and it is therefore not surprising that they are also responsible for a disproportionate amount of healthcare AI research for DR in LMICs. Additionally, China’s and India’s middle-income (as opposed to low-income) status and more developed technology sectors has meant that there is greater in-country expertise and technology infrastructure to facilitate the development and testing of healthcare AI.^70^

However, if AI for DR and other conditions is to be implemented and used to reduce healthcare disparities, as is often suggested,^71^ it is essential that contextually relevant research around the use of AI in clinical pathways is done in less well-resourced regions of the world. Datasets from populations in LMICs that are used to train AI models are necessary to prevent what has been coined ‘health data poverty’,^13^ whereby populations in poorer regions of the world, as well as minority ethnic groups in high-income countries, are disadvantaged through a lack of training data from such populations.

If these issues are not considered and addressed, global health inequities could be further exaggerated with wealthier countries that have invested in healthcare AI having access to, and using, new technologies and poorer countries left behind.

A further consideration is that screening for DR is only one part of a larger program that is required to reduce avoidable sight loss from DR. Improved access to retinal laser treatments and antivascular endothelial growth factor drugs is required with adequately trained eye care staff more widely available to deliver these treatments, particularly in low-resource settings. Without good access to affordable treatments that can be delivered effectively, improved screening for DR will not reduce sight loss from the disease.

However, before any of this becomes reality, healthcare AI needs to be integrated and used in clinical pathways. The current literature around AI for DR in LMICs has largely focused on AI’s performance in terms of sensitivities and specificities and does not adequately address the complex process of integrating this new technology into clinical care—a process which is likely to be even more challenging in LMICs.

We identified only three studies that focused on the implementation of an AI tool into an active clinical pathway in an LMIC^46 47^; potential benefits included improved rates of follow-up following a point-of-care referral decision,^18^ although several challenges were also highlighted. The majority of studies described the development of AI models and only just over half were considered external validations. Of those AI tools that had been externally validated, we identified commercial websites for Medios AI, SELENA+, EyeArt, RAIDS and EyeWisdom and a non-commercial (charity) website for Cybersight, suggesting only some of the identified AI tools are ready for clinical deployment.

Few studies stated whether their AI tool had any regulatory approvals (eg, FDA or CE marking) and there is almost no available information on the cost of such systems, either in the literature or on commercial websites. These are all critical factors when hospitals and/or policy makers are deciding on whether to use AI in clinical care.^72^

Moreover, a likely major advantage of healthcare AI for LMICs, as well as high-income countries, will be the potential health economic gains. We identified two economic evaluation studies, one of which concluded that AI for DR screening in Brazil was not cost-effective. The lack of transparency around the cost of AI systems makes such analyses difficult.

As we have highlighted, Google has a large portfolio of research around using AI for DR in LMICs, including one of only three studies that looked specifically at the implementation of AI for DR in an active clinical pathway.^46^ This paper candidly described the difficulties the team had and highlights the importance of implementation research embedded within prospective studies.

The requirement for a good internet connection to run their AI model was particularly highlighted as impractical. Other countries considering using AI that do not have access to a reliable and fast internet connection may face similar difficulties, suggesting models that can run on isolated machines offline may be more appropriate.

Despite Google’s large portfolio of research including implementation considerations, it should be noted that all of Google’s published work in Thailand and India was funded by Google with the majority of authors either employed by, or consultants for, Google. Additionally, there is no indication in any publication that Google’s AI system is or will be made available for use in clinical practice. If indeed the research is done with the intention of improving eye care, more transparency about access to and use of Google’s technology would be welcome.

In addition to the aforementioned clinical and implementation challenges, there are myriad legal and ethical issues adding further complexity which have not been addressed. For example, there are questions around accountability if errors are made, and legal frameworks for managing patient imaging and clinical data are needed,^72^ although ethical considerations were beyond the scope of this review.

Investment in hardware infrastructure in LMICs that would enable patient data to be hosted on servers in the country where the AI is being used, for example, would provide a higher degree of control over data to the institutions and host countries, and would help with the curation of locally representative datasets, thereby addressing the issue of ‘health data poverty’.^13^ Research programs investing in healthcare AI in LMICs have an opportunity to contribute to this, especially if work is done in collaboration with Ministries of Health.

The performance of the AI systems identified in this review demonstrates the potential for AI to improve diabetic eye care services in LMICs. There is a real opportunity for the quality of health service delivery in LMICs to be rapidly improved through leveraging such technologies. However, further research simply publishing the performance of AI tools in terms of sensitivities and specificities will not help this become reality.

The focus needs to move towards integrating AI models into health systems and detailing if and how their use improves clinical practice. Of the 81 studies included as full texts in this review, we identified only 1 randomized controlled trial. As AI tools are medical devices, it is important that, where possible, there is prospective clinical trial evidence to measure the effect of AI on clinical care prior to wider implementation. Clearer reporting of the impact of ungradable images on AI performance would also improve the evidence base.

Implementation research investigating how such systems can most effectively integrate into clinical pathways is needed as well as qualitative research specifically around acceptability and fidelity and LMIC population-specific dataset curation. As the two economic evaluations identified in this review demonstrate it is unclear whether using AI for DR screening is cost-effective, further work is needed in this regard.

The primary focus of the majority of studies identified in this review was sensitivity and specificity of the respective AI system to grade DR. However, no publication directly compared more than one AI model, therefore making it very difficult to compare the performance of different AI tools. Future validation work directly comparing different AI systems on the same image dataset by independent investigators would be of significant value and would enable a better comparison of performance. However, any commercially available AI systems included in such work should not be anonymized, otherwise comparative performance data would be of limited value.

If all this can be done, LMICs will be better placed to benefit from ongoing healthcare technology developments and, through the curation of LMIC population-specific datasets, will be able to maximize the performance of AI models in their populations. The potential of healthcare AI for DR as well as other conditions is arguably greatest in poorer regions of the world where there are fewer clinicians; however, there are a number of challenges to overcome if this potential is to be translated into reality.

## Data Availability

Data sharing not applicable as no datasets generated and/or analyzed for this study.
