# Supplementary material for: Artificial intelligence for diabetic retinopathy in low-income and middle-income countries: a scoping review
Source: BMJ Open Diabetes Res Care. 2023 Aug 2;11(4):e003424. doi: 10.1136/bmjdrc-2023-003424 (PMC10401245; doi:10.1136/bmjdrc-2023-003424)
Supplement: Supplementary data [file bmjdrc-2023-003424supp001.pdf]

Search strategies

MEDLINE

1. artificial intelligence/
2. deep learning/
3. exp machine learning/
4. "neural networks (computer)"/
5. fuzzy logic/
6. algorithms/
7. decision tree/
8. automation/
9. databases, factual/
10. information processing/
11. (artificial adj1 intelligence).tw.
12. ((deep or machine or transfer) adj2 learning).tw.
13. (vector adj3 machine).tw.
14. (AI or DLS).tw.
15. ((deep or convolutional or neural) adj3 network$).tw.
16. (automat$ adj2 (screen$ or detect$ or diagnos$ or algorithm$ or identif$ or grading or graded or method$)).tw.
17. Image Interpretation, Computer-Assisted/
18. (computer adj2 aid$ adj2 diagnos$).tw.
19. (EfficientNet adj1 B3).tw.
20. (AlexNet or DenseNet or GoogLeNet or MobileNet or NASNet or ResNet or Ensemble or Inception-v3 VGG16 or Xception).tw.
21. (Neural adj1 Network adj1 Classifier$).tw.
22. (segmentation adj2 (imag$ or network$)).tw.
23. (capsule adj1 network$).tw.
24. (multi-label adj1 classificat$).tw.
25. (Multiple adj1 Lesion$ adj1 Insert$).tw.
26. (Multiple adj1 Instance adj1 Learn$).tw.
27. Bagging.tw.
28. (Naive adj1 Bayes).tw.
29. (Multilayer adj1 Perceptron).tw.
30. ((multi-layer adj1 perceptron) or MLP).tw.
31. (Radial adj1 Basis adj1 Function).tw.
32. (Random adj1 Forest).tw.
33. ((Ada or gradient) adj1 boost$).tw.
34. LASSO.tw.
35. (Elastic adj1 Net).tw.
36. (genetic adj1 algorithm$).tw.
37. ((decision or classification or regression or probability or model$) adj3 tree$).tw.

38. (logistic$ adj2 regression adj15 learn$).tw.
39. (augment$ adj1 clinical adj1 decision$ adj1 mak$).tw.
40. (nearest adj1 (neighbor or neighbour)).tw.
41. (fuzzy adj3 (logit or logic or logistic)).tw.
42. kernel.tw.
43. Datasets as Topic/
44. (Kaggle or EyePACS or Messidor or DIARETDB1 or DIARETDB0 or e-Ophtha or UoA-DR or IDRiD or Ophdiat or HEI-MED or DiaretDB or APTOS-2019).tw.
45. or/1-44
46. Diabetic Retinopathy/
47. ((diabet$ or proliferative or non-proliferative) adj4 retinopath$).tw.
48. diabetic retinopathy.kw.
49. (diabet$ adj3 (eye$ or vision or visual$ or sight$)).tw.
50. (retinopath$ adj3 (eye$ or vision or visual$ or sight$)).tw.
51. (DR adj3 (eye$ or vision or visual$ or sight$)).tw.
52. or/46-51
53. 45 and 52
54. limit 53 to (comment or editorial or letter)
55. 53 not 54
56. (prematurity or glucose or glycemic or sleep).ti.
57. 55 not 56
58. Developing Countries/
59. ((developing or less* developed or under developed or underdeveloped or middle income or low* income) adj (economy or economies)).ti,ab.
60. ((developing or less* developed or under developed or underdeveloped or middle income or low* income or underserved or under served or deprived or poor*) adj (countr* or nation? or population? or world)).ti,ab.
61. (low* adj (gdp or gnp or gross domestic or gross national)).ti,ab.
62. (low adj3 middle adj3 countr*).ti,ab.
63. (lmic or lmics or third world or lami countr*).ti,ab.
64. transitional countr*.ti,ab.
65. global south.ti,ab.
66. "africa south of the sahara"/
67. africa, central/
68. africa, eastern/
69. africa, southern/
70. africa, western/
71. ("Africa South of the Sahara" or sub-Saharan Africa or subSaharan Africa).ti,ab.
72. Central Africa.ti,ab.
73. Eastern Africa.ti,ab.
74. Southern Africa.ti,ab.
75. Western Africa.ti,ab.
76. "Democratic People's Republic of Korea"/

77. (North Korea or (Democratic People* Republic adj2 Korea)).ti,ab.
78. Cambodia/
79. Cambodia.ti,ab.
80. Indonesia/
81. (Indonesia or Dutch East Indies).ti,ab.
82. (Kiribati or Gilbert Islands or Phoenix Islands or Line Islands).ti,ab.
83. Laos/
84. (Laos or (Lao adj1 Democratic Republic)).ti,ab.
85. Micronesia/
86. Micronesia.ti,ab.
87. Mongolia/
88. Mongolia.ti,ab.
89. Myanmar/
90. (Myanmar or Burma).ti,ab.
91. Papua New Guinea/
92. (Papua New Guinea or German New Guinea or British New Guinea or Territory of Papua).ti,ab.
93. Philippines/
94. (Philippines or Philippine Islands).ti,ab.
95. "Independent State of Samoa"/
96. ((Samoa not American Samoa) or Western Samoa or Navigator Islands or Samoan Islands).ti,ab.
97. Solomon Islands.ti,ab.
98. Timor-Leste/
99. (Timor-Leste or East Timor or Portuguese Timor).ti,ab.
100. Vanuatu/
101. (Vanuatu or New Hebrides).ti,ab.
102. Vietnam/
103. (Viet Nam or Vietnam or French Indochina).ti,ab.
104. American Samoa/
105. American Samoa.ti,ab.
106. exp China/
107. China.ti,ab.
108. Fiji/
109. Fiji.ti,ab.
110. Malaysia/
111. (Malaysia or Malayan Union or Malaya).ti,ab.
112. Marshall Islands.ti,ab.
113. Nauru.ti,ab.
114. Thailand/
115. (Thailand or Siam).ti,ab.
116. Tonga/
117. Tonga.ti,ab.
118. (Tuvalu or Ellice Islands).ti,ab.

119. Kyrgyzstan/
120. (Kyrgyzstan or Kyrgyz Republic or Kirghizia or Kirghiz).ti,ab.
121. Tajikistan/
122. Tajikistan.ti,ab.
123. Ukraine/
124. Ukraine.ti,ab.
125. Uzbekistan/
126. Uzbekistan.ti,ab.
127. Albania/
128. Albania.ti,ab.
129. Armenia/
130. Armenia.ti,ab.
131. Azerbaijan/
132. Azerbaijan.ti,ab.
133. "Republic of Belarus"/
134. (Belarus or Byelarus or Byelorussia or Belorussia).ti,ab.
135. Bosnia-Herzegovina/
136. (Bosnia or Herzegovina).ti,ab.
137. Bulgaria/
138. Bulgaria.ti,ab.
139. "Georgia (Republic)"/
140. Georgia.ti,ab. not Georgia/
141. Kazakhstan/
142. (Kazakhstan or Kazakh).ti,ab.
143. Kosovo/
144. Kosovo.ti,ab.
145. Moldova/
146. Moldova.ti,ab.
147. Montenegro/
148. Montenegro.ti,ab.
149. "Republic of North Macedonia"/
150. North Macedonia.ti,ab.
151. Romania/
152. Romania.ti,ab.
153. exp Russia/
154. "Russia (Pre-1917)"/
155. USSR/
156. (Russia or Russian Federation or USSR or Union of Soviet Socialist Republics or Soviet Union).ti,ab.
157. Serbia/
158. Serbia.ti,ab.
159. Turkey/

160. (Turkey.ti,ab. not animal/) or (Anatolia or Asia Minor).ti,ab.
161. Turkmenistan/
162. Turkmenistan.ti,ab.
163. Belize/
164. (Belize or British Honduras).ti,ab.
165. Bolivia/
166. Bolivia.ti,ab.
167. El Salvador/
168. El Salvador.ti,ab.
169. Haiti/
170. (Haiti or Hayti).ti,ab.
171. Honduras/
172. Honduras.ti,ab.
173. Nicaragua/
174. Nicaragua.ti,ab.
175. Argentina/
176. (Argentina or Argentine Republic).ti,ab.
177. Brazil/
178. Brazil.ti,ab.
179. Colombia/
180. Colombia.ti,ab.
181. Costa Rica/
182. Costa Rica.ti,ab.
183. Cuba/
184. Cuba.ti,ab.
185. Dominica/
186. Dominica.ti,ab.
187. Dominican Republic/
188. Dominican Republic.ti,ab.
189. Ecuador/
190. Ecuador.ti,ab.
191. Grenada/
192. Grenada.ti,ab.
193. Guatemala/
194. Guatemala.ti,ab.
195. Guyana/
196. (Guyana or British Guiana).ti,ab.
197. Jamaica/
198. Jamaica.ti,ab.
199. Mexico/
200. (Mexico or United Mexican States).ti,ab.
201. Panama/

202. Panama.ti,ab.
203. Paraguay/
204. Paraguay.mp.
205. Peru/
206. Peru.ti,ab.
207. Saint Lucia/
208. (St Lucia or Saint Lucia or Iyonala or Hewanorra).ti,ab.
209. "Saint Vincent and the Grenadines"/
210. (Saint Vincent or St Vincent or Grenadines).ti,ab.
211. Suriname/
212. (Suriname or Dutch Guiana).ti,ab.
213. Venezuela/
214. Venezuela.ti,ab.
215. Algeria/
216. Algeria.ti,ab.
217. Djibouti/
218. (Djibouti or French Somaliland).ti,ab.
219. Egypt/
220. Egypt.ti,ab.
221. Iran/
222. (Iran or Persia).ti,ab.
223. Morocco/
224. Morocco.ti,ab.
225. Tunisia/
226. Tunisia.mp.
227. (Gaza or West Bank or Palestine).ti,ab.
228. Iraq/
229. (Iraq or Mesopotamia).ti,ab.
230. Jordan/
231. Jordan.ti,ab.
232. Lebanon/
233. (Lebanon or Lebanese Republic).ti,ab.
234. Libya/
235. Libya.ti,ab.
236. Afghanistan/
237. Afghanistan.ti,ab.
238. Bangladesh/
239. Bangladesh.ti,ab.
240. Bhutan/
241. Bhutan.ti,ab.
242. exp India/
243. India.ti,ab.

244. Nepal/
245. Nepal.ti,ab.
246. Pakistan/
247. Pakistan.ti,ab.
248. Sri Lanka/
249. (Sri Lanka or Ceylon).ti,ab.
250. Maldives.ti,ab. [UPPER MIDDLE INCOME COUNTRIES IN SOUTH ASIA]
251. Angola/
252. Angola.ti,ab.
253. Benin/
254. Benin.ti,ab.
255. Cameroon/
256. (Cameroon or Kamerun or Cameroun).ti,ab.
257. Cape Verde/
258. (Cape Verde or Cabo Verde).ti,ab.
259. Comoros/
260. (Comoros or Glorioso Islands or Mayotte).ti,ab.
261. Congo/
262. (Congo not ((Democratic Republic adj3 Congo) or congo red or crimean-congo)).ti,ab.
263. Cote d'Ivoire/
264. (Cote d'Ivoire or Cote dIvoire or Ivory Coast).ti,ab.
265. Eswatini/
266. (eSwatini or Swaziland).ti,ab.
267. Ghana/
268. (Ghana or Gold Coast).ti,ab.
269. Kenya/
270. (Kenya or East Africa Protectorate).ti,ab.
271. Lesotho/
272. (Lesotho or Basutoland).ti,ab.
273. Mauritania/
274. Mauritania.ti,ab.
275. Nigeria/
276. Nigeria.ti,ab.
277. (Sao Tome adj2 Principe).ti,ab.
278. Senegal/
279. Senegal.ti,ab.
280. Tanzania/
281. (Tanzania or Tanganyika or Zanzibar).ti,ab.
282. Zambia/
283. (Zambia or Northern Rhodesia).ti,ab.
284. Zimbabwe/
285. (Zimbabwe or Southern Rhodesia).ti,ab.

286. Botswana/
287. (Botswana or Bechuanaland or Kalahari).ti,ab.
288. Equatorial Guinea/
289. (Equatorial Guinea or Spanish Guinea).ti,ab.
290. Gabon/
291. (Gabon or Gabonese Republic).ti,ab.
292. Mauritius/
293. (Mauritius or Agalega Islands).ti,ab.
294. Namibia/
295. (Namibia or German South West Africa).ti,ab.
296. South Africa/
297. (South Africa or Cape Colony or British Bechuanaland or Boer Republics or Zululand or Transvaal or Natalia Republic or Orange Free State).ti,ab.
298. Syria/
299. (Syria or Syrian Arab Republic).ti,ab.
300. Yemen/
301. Yemen.ti,ab.
302. Burkina Faso/
303. (Burkina Faso or Burkina Fasso or Upper Volta).ti,ab.
304. Burundi/
305. (Burundi or Ruanda-Urundi).ti,ab.
306. Central African Republic/
307. (Central African Republic or Ubangi-Shari).ti,ab.
308. Chad/
309. Chad.ti,ab.
310. "Democratic Republic of the Congo"/
311. (((Democratic Republic or DR) adj2 Congo) or Congo-Kinshasa or Belgian Congo or Zaire or Congo Free State).ti,ab.
312. Eritrea/
313. Eritrea.ti,ab.
314. Ethiopia/
315. (Ethiopia or Abyssinia).ti,ab.
316. Gambia/
317. Gambia.ti,ab.
318. Guinea/
319. (Guinea not (New Guinea or Guinea Pig* or Guinea Fowl or Guinea-Bissau or Portuguese Guinea or Equatorial Guinea)).ti,ab.
320. Guinea-Bissau/
321. (Guinea-Bissau or Portuguese Guinea).ti,ab.
322. Liberia/
323. Liberia.ti,ab.
324. Madagascar/
325. (Madagascar or Malagasy Republic).ti,ab.

326. Malawi/
327. (Malawi or Nyasaland).ti,ab.
328. Mali/
329. Mali.ti,ab.
330. Mozambique/
331. (Mozambique or Mocambique or Portuguese East Africa).ti,ab.
332. Niger/
333. (Niger not (Aspergillus or Peptococcus or Schizothorax or Cruciferae or Gobius or Lasius or Agelastes or Melanosuchus or radish or Parastromateus or Orius or Apergillus or Parastromateus or Stomoxys)).ti,ab.
334. Rwanda/
335. (Rwanda or Ruanda).ti,ab.
336. Sierra Leone/
337. (Sierra Leone or Salone).ti,ab.
338. Somalia/
339. (Somalia or Somaliland).ti,ab.
340. South Sudan/
341. South Sudan.ti,ab.
342. Sudan/
343. Sudan.ti,ab.
344. Togo/
345. (Togo or Togolese Republic or Togoland).ti,ab.
346. Uganda/
347. Uganda.ti,ab.
348. or/58-347 [ALL LMICs]
349. 57 and 348

Embase

1. artificial intelligence/
2. deep learning/
3. machine learning/
4. supervised machine learning/ or support vector machine/ or unsupervised machine learning/
5. perceptron/
6. artificial neural network/
7. convolutional neural network/
8. deep neural network/
9. automated pattern recognition/
10. decision tree/
11. detection algorithm/
12. learning algorithm/

13. classification algorithm/
14. data classification/
15. disease classification/
16. disease simulation/
17. automation/
18. information processing/
19. feature extraction/
20. bayesian learning/
21. fuzzy system/
22. k nearest neighbor/
23. kernel method/
24. random forest/
25. (artificial adj1 intelligence).tw.
26. ((deep or machine or transfer) adj2 learning).tw.
27. (vector adj3 machine).tw.
28. (AI or DLS).tw.
29. ((deep or convolutional or neural) adj3 network$).tw.
30. (automat$ adj2 (screen$ or detect$ or diagnos$ or algorithm$ or identif$ or grading or graded or method$)).tw.
31. (computer adj2 aid$ adj2 diagnos$).tw.
32. (EfficientNet adj1 B3).tw.
33. (AlexNet or DenseNet or GoogLeNet or MobileNet or NASNet or ResNet or Ensemble or Inception-v3 VGG16 or Xception).tw.
34. (Neural adj1 Network adj1 Classifier$).tw.
35. (segmentation adj2 (imag$ or network$)).tw.
36. (capsule adj1 network$).tw.
37. (multi-label adj1 classificat$).tw.
38. (Multiple adj1 Lesion$ adj1 Insert$).tw.
39. (Multiple adj1 Instance adj1 Learn$).tw.
40. Bagging.tw.
41. (Naive adj1 Bayes).tw.
42. (Multilayer adj1 Perceptron).tw.
43. ((multi-layer adj1 perceptron) or MLP).tw.
44. (Radial adj1 Basis adj1 Function).tw.
45. (Random adj1 Forest).tw.
46. ((Ada or gradient) adj1 boost$).tw.
47. LASSO.tw.
48. (Elastic adj1 Net).tw.
49. (genetic adj1 algorithm$).tw.
50. ((decision or classification or regression or probability or model$) adj3 tree$).tw.
51. (logistic$ adj2 regression adj15 learn$).tw.
52. (augment$ adj1 clinical adj1 decision$ adj1 mak$).tw.

53. (nearest adj1 (neighbor or neighbour)).tw.
54. (fuzzy adj3 (logit or logic or logistic)).tw.
55. kernel.tw.
56. computer assisted diagnosis/
57. (Kaggle or EyePACS or Messidor or DIARETDB1 or DIARETDB0 or e-Ophtha or UoA-DR or IDRiD or Ophdiat or HEI-MED or DiaretDB or APTOS-2019).tw.
58. or/1-57
59. exp diabetic retinopathy/
60. ((diabet$ or proliferative or non-proliferative) adj4 retinopath$).tw.
61. diabetic retinopathy.kw.
62. (diabet$ adj3 (eye$ or vision or visual$ or sight$)).tw.
63. (retinopath$ adj3 (eye$ or vision or visual$ or sight$)).tw.
64. (DR adj3 (eye$ or vision or visual$ or sight$)).tw.
65. or/59-64
66. 58 and 65
67. (prematurity or glucose or glycemic or sleep).ti.
68. 66 not 67
69. developing country/ or low income country/ or middle income country/
70. ((developing or less* developed or under developed or underdeveloped or middle income or low* income) adj (economy or economies)).ti,ab.
71. ((developing or less* developed or under developed or underdeveloped or middle income or low* income or underserved or under served or deprived or poor*) adj (countr* or nation? or population? or world)).ti,ab.
72. (low* adj (gdp or gnp or gross domestic or gross national)).ti,ab.
73. (low adj3 middle adj3 countr*).ti,ab.
74. (lmic or lmics or third world or lami countr*).ti,ab.
75. transitional countr*.ti,ab.
76. global south.ti,ab.
77. "Africa south of the Sahara"/
78. ("Africa South of the Sahara" or sub-Saharan Africa or subSaharan Africa).ti,ab.
79. Central Africa.ti,ab.
80. Eastern Africa.ti,ab.
81. Southern Africa.ti,ab.
82. Western Africa.ti,ab.
83. North Korea/
84. (North Korea or (Democratic People* Republic adj2 Korea)).ti,ab.
85. Haiti/
86. (Haiti or Hayti).ti,ab.
87. Afghanistan/
88. Afghanistan.ti,ab.
89. Nepal/
90. Nepal.ti,ab.
91. Syrian Arab Republic/

92. (Syria or Syrian Arab Republic).ti,ab.
93. Yemen/
94. Yemen.ti,ab.
95. Tajikistan/
96. Tajikistan.ti,ab.
97. Benin/
98. (Benin or Dahomey).ti,ab.
99. Burkina Faso/
100. (Burkina Faso or Burkina Fasso or Upper Volta).ti,ab.
101. Burundi/
102. (Burundi or Ruanda-Urundi).ti,ab.
103. Central African Republic/
104. (Central African Republic or Ubangi-Shari).ti,ab.
105. Chad/
106. Chad.ti,ab.
107. Democratic Republic Congo/
108. (((Democratic Republic or DR) adj2 Congo) or Congo-Kinshasa or Belgian Congo or Zaire or Congo Free State).ti,ab.
109. Eritrea/
110. Eritrea.ti,ab.
111. Ethiopia/
112. (Ethiopia or Abyssinia).ti,ab.
113. Gambia/
114. Gambia.ti,ab.
115. Guinea/
116. (Guinea not (New Guinea or Guinea Pig* or Guinea Fowl or Guinea-Bissau or Portuguese Guinea or Equatorial Guinea)).ti,ab.
117. Guinea-Bissau/
118. (Guinea-Bissau or Portuguese Guinea).ti,ab.
119. Liberia/
120. Liberia.ti,ab.
121. Madagascar/
122. (Madagascar or Malagasy Republic).ti,ab.
123. Malawi/
124. (Malawi or Nyasaland).ti,ab.
125. Mali/
126. Mali.ti,ab.
127. Mozambique/
128. (Mozambique or Mocambique or Portuguese East Africa).ti,ab.
129. Niger/
130. (Niger not (Aspergillus or Peptococcus or Schizothorax or Cruciferae or Gobius or Lasius or Agelastes or Melanosuchus or radish or Parastromateus or Orius or Apergillus or Parastromateus or Stomoxys)).ti,ab.

131. Rwanda/
132. (Rwanda or Ruanda).ti,ab.
133. Sierra Leone/
134. (Sierra Leone or Salone).ti,ab.
135. Somalia/
136. (Somalia or Somaliland).ti,ab.
137. south sudan/
138. South Sudan.ti,ab.
139. Tanzania/
140. (Tanzania or Tanganyika or Zanzibar).ti,ab.
141. Togo/
142. (Togo or Togolese Republic or Togoland).ti,ab.
143. Uganda/
144. Uganda.ti,ab.
145. Cambodia/
146. Cambodia.ti,ab.
147. exp Indonesia/
148. (Indonesia or Dutch East Indies).ti,ab.
149. kiribati/
150. (Kiribati or Gilbert Islands or Phoenix Islands or Line Islands).ti,ab.
151. Laos/
152. (Laos or (Lao adj1 Democratic Republic)).ti,ab.
153. exp "Federated States of Micronesia"/
154. Micronesia.ti,ab.
155. Mongolia/
156. Mongolia.ti,ab.
157. Myanmar/
158. (Myanmar or Burma).ti,ab.
159. Papua New Guinea/
160. (Papua New Guinea or German New Guinea or British New Guinea or Territory of Papua).ti,ab.
161. Philippines/
162. (Philippines or Philippine Islands).ti,ab.
163. solomon islands/
164. Solomon Islands.ti,ab.
165. Timor-Leste/
166. (Timor-Leste or East Timor or Portuguese Timor).ti,ab.
167. Vanuatu/
168. (Vanuatu or New Hebrides).ti,ab.
169. Viet Nam/
170. (Viet Nam or Vietnam or French Indochina).ti,ab.
171. Kyrgyzstan/
172. (Kyrgyzstan or Kyrgyz Republic or Kirghizia or Kirghiz).ti,ab.

173. Moldova/
174. Moldova.ti,ab.
175. exp Ukraine/
176. Ukraine.ti,ab.
177. exp Uzbekistan/
178. Uzbekistan.ti,ab.
179. Bolivia/
180. Bolivia.ti,ab.
181. El Salvador/
182. El Salvador.ti,ab.
183. Honduras/
184. Honduras.ti,ab.
185. Nicaragua/
186. Nicaragua.ti,ab.
187. Djibouti/
188. (Djibouti or French Somaliland).ti,ab.
189. Egypt/
190. Egypt.ti,ab.
191. Morocco/
192. Morocco.ti,ab.
193. Tunisia/
194. Tunisia.mp.
195. palestine/
196. (Gaza or West Bank or Palestine).ti,ab.
197. Bangladesh/
198. Bangladesh.ti,ab.
199. Bhutan/
200. Bhutan.ti,ab.
201. exp India/
202. India.ti,ab.
203. exp Pakistan/
204. Pakistan.ti,ab.
205. Angola/
206. Angola.ti,ab.
207. Cameroon/
208. (Cameroon or Kamerun or Cameroun).ti,ab.
209. Cape Verde/
210. (Cape Verde or Cabo Verde).ti,ab.
211. Comoros/
212. (Comoros or Glorioso Islands or Mayotte).ti,ab.
213. Congo/
214. (Congo not ((Democratic Republic adj3 Congo) or congo red or crimean-congo)).ti,ab.

215. Cote d'Ivoire/
216. (Cote d'Ivoire or Cote dIvoire or Ivory Coast).ti,ab.
217. eswatini/
218. (eSwatini or Swaziland).ti,ab.
219. Ghana/
220. (Ghana or Gold Coast).ti,ab.
221. Kenya/
222. (Kenya or East Africa Protectorate).ti,ab.
223. Lesotho/
224. (Lesotho or Basutoland).ti,ab.
225. Mauritania/
226. Mauritania.ti,ab.
227. Nigeria/
228. Nigeria.ti,ab.
229. "sao tome and principe"/
230. (Sao Tome adj2 Principe).ti,ab.
231. Senegal/
232. Senegal.ti,ab.
233. Sudan/
234. (Sudan not South Sudan).ti,ab.
235. Zambia/
236. (Zambia or Northern Rhodesia).ti,ab.
237. Zimbabwe/
238. (Zimbabwe or Southern Rhodesia).ti,ab.
239. American Samoa/
240. American Samoa.ti,ab.
241. china/ or guangxi/ or inner mongolia/ or macao/ or ningxia/ or tibet/ or xinjiang/
242. China.ti,ab.
243. Fiji/
244. Fiji.ti,ab.
245. exp Malaysia/
246. (Malaysia or Malayan Union or Malaya).ti,ab.
247. marshall islands/
248. Marshall Islands.ti,ab.
249. nauru/
250. Nauru.ti,ab.
251. Samoa/
252. ((Samoa not American Samoa) or Western Samoa or Navigator Islands or Samoan Islands).ti,ab.
253. Thailand/
254. (Thailand or Siam).ti,ab.
255. Tonga/
256. Tonga.ti,ab.

257. tuvalu/
258. (Tuvalu or Ellice Islands).ti,ab.
259. Albania/
260. Albania.ti,ab.
261. Armenia/
262. Armenia.ti,ab.
263. exp Azerbaijan/
264. Azerbaijan.ti,ab.
265. Belarus/
266. (Belarus or Byelarus or Byelorussia or Belorussia).ti,ab.
267. exp "Bosnia and Herzegovina"/
268. (Bosnia or Herzegovina).ti,ab.
269. Bulgaria/
270. Bulgaria.ti,ab.
271. exp "Georgia (republic)"/
272. Georgia.ti,ab. not "georgia (u.s.)"/
273. Kazakhstan/
274. (Kazakhstan or Kazakh).ti,ab.
275. Kosovo/
276. Kosovo.ti,ab.
277. "Montenegro (republic)"/
278. Montenegro.ti,ab.
279. "republic of north macedonia"/
280. North Macedonia.ti,ab.
281. Romania/
282. Romania.ti,ab.
283. exp Russian Federation/
284. ussr/
285. (Russia or Russian Federation or USSR or Union of Soviet Socialist Republics or Soviet Union).ti,ab.
286. exp Serbia/
287. Serbia.ti,ab.
288. "Turkey (republic)"/
289. (Turkey.ti,ab. not "Turkey (bird)"/) or (Anatolia or Asia Minor).ti,ab.
290. Turkmenistan/
291. Turkmenistan.ti,ab.
292. Argentina/
293. (Argentina or Argentine Republic).ti,ab.
294. Belize/
295. (Belize or British Honduras).ti,ab.
296. exp Brazil/
297. Brazil.ti,ab.

298. Colombia/
299. Colombia.ti,ab.
300. Costa Rica/
301. Costa Rica.ti,ab.
302. Cuba/
303. Cuba.ti,ab.
304. Dominica/
305. Dominica.ti,ab.
306. Dominican Republic/
307. Dominican Republic.ti,ab.
308. Ecuador/
309. Ecuador.ti,ab.
310. Grenada/
311. Grenada.ti,ab.
312. Guatemala/
313. Guatemala.ti,ab.
314. Guyana/
315. (Guyana or British Guiana).ti,ab.
316. Jamaica/
317. Jamaica.ti,ab.
318. exp Mexico/
319. (Mexico or United Mexican States).ti,ab.
320. Paraguay/
321. Paraguay.mp.
322. Peru/
323. Peru.ti,ab.
324. Saint Lucia/
325. (St Lucia or Saint Lucia or Iyonala or Hewanorra).ti,ab.
326. "Saint Vincent and the Grenadines"/
327. (Saint Vincent or St Vincent or Grenadines).ti,ab.
328. Suriname/
329. (Suriname or Dutch Guiana).ti,ab.
330. Venezuela/
331. Venezuela.ti,ab.
332. Algeria/
333. Algeria.ti,ab.
334. Iran/
335. (Iran or Persia).ti,ab.
336. exp Iraq/
337. (Iraq or Mesopotamia).ti,ab.
338. Jordan/
339. Jordan.ti,ab.

340. Lebanon/
341. (Lebanon or Lebanese Republic).ti,ab.
342. Libyan Arab Jamahiriya/
343. libya.ti,ab.
344. maldives/
345. Maldives.ti,ab.
346. Sri Lanka/
347. (Sri Lanka or Ceylon).ti,ab.
348. Botswana/
349. (Botswana or Bechuanaland or Kalahari).ti,ab.
350. Equatorial Guinea/
351. (Equatorial Guinea or Spanish Guinea).ti,ab.
352. Gabon/
353. (Gabon or Gabonese Republic).ti,ab.
354. Mauritius/
355. (Mauritius or Agalega Islands).ti,ab.
356. Namibia/
357. (Namibia or German South West Africa).ti,ab.
358. South Africa/
359. (South Africa or Cape Colony or British Bechuanaland or Boer Republics or Zululand or Transvaal or Natalia Republic or Orange Free State).ti,ab.
360. or/69-359 [ALL LOW AND MIDDLE-INCOME COUNTRIES]
361. 68 and 360

Global Health

1. neural networks/
2. fuzzy logic/
3. algorithms/
4. automation/
5. data processing/
6. datasets/
7. image processing/
8. learning/
9. learning ability/
10. (artificial adj1 intelligence).tw.
11. ((deep or machine or transfer) adj2 learning).tw.
12. (vector adj3 machine).tw.
13. (AI or DLS).tw.
14. ((deep or convolutional or neural) adj3 network$).tw.
15. (automat$ adj2 (screen$ or detect$ or diagnos$ or algorithm$ or identif$ or grading or graded or method$)).tw.

16. (computer adj2 aid$ adj2 diagnos$).tw.
17. (EfficientNet adj1 B3).tw.
18. (AlexNet or DenseNet or GoogLeNet or MobileNet or NASNet or ResNet or Inception-v3 VGG16 or Xception).tw.
19. (Neural adj1 Network adj1 Classifier$).tw.
20. (segmentation adj2 (imag$ or network$)).tw.
21. (capsule adj1 network$).tw.
22. (multi-label adj1 classificat$).tw.
23. (Multiple adj1 Lesion$ adj1 Insert$).tw.
24. (Multiple adj1 Instance adj1 Learn$).tw.
25. Bagging.tw.
26. (Naive adj1 Bayes).tw.
27. (Multilayer adj1 Perceptron).tw.
28. ((multi-layer adj1 perceptron) or MLP).tw.
29. (Radial adj1 Basis adj1 Function).tw.
30. (Random adj1 Forest).tw.
31. ((Ada or gradient) adj1 boost$).tw.
32. LASSO.tw.
33. (Elastic adj1 Net).tw.
34. (genetic adj1 algorithm$).tw.
35. ((decision or classification or regression or probability or model$) adj3 tree$).tw.
36. (logistic$ adj2 regression adj15 learn$).tw.
37. (augment$ adj1 clinical adj1 decision$ adj1 mak$).tw.
38. (nearest adj1 (neighbor or neighbour)).tw.
39. (fuzzy adj3 (logit or logic or logistic)).tw.
40. kernel.tw.
41. (Kaggle or EyePACS or Messidor or DIARETDB1 or DIARETDB0 or e-Ophtha or UoA-DR or IDRiD or Ophdiat or HEI-MED or DiaretDB or APTOS-2019).tw.
42. or/1-41
43. ((diabet$ or proliferative or non-proliferative) adj4 retinopath$).tw.
44. (diabet$ adj3 (eye$ or vision or visual$ or sight$)).tw.
45. (retinopath$ adj3 (eye$ or vision or visual$ or sight$)).tw.
46. (DR adj3 (eye$ or vision or visual$ or sight$)).tw.
47. or/43-46
48. 42 and 47
49. exp oecd countries/
50. exp high human development index countries/ or exp high income countries/ or exp very high human development index countries/
51. or/49-50
52. 48 not 51

CENTRAL on the Cochrane Library

#1	MeSH descriptor: [Artificial Intelligence] this term only
#2	MeSH descriptor: [Deep Learning] this term only
#3	MeSH descriptor: [Machine Learning] explode all trees
#4	MeSH descriptor: [Neural Networks, Computer] this term only
#5	MeSH descriptor: [Algorithms] this term only
#6	MeSH descriptor: [Decision Trees] this term only
#7	MeSH descriptor: [Automation] this term only
#8	MeSH descriptor: [Databases, Factual] this term only
#9	MeSH descriptor: [Electronic Data Processing] this term only
#10	artificial NEAR/1 intelligence
#11	(deep or machine or transfer) NEAR/2 learning
#12	vector NEAR/3 machine
#13	AI or DLS
#14	(deep or convolutional or neural) NEAR/3 network*
#15	automat* NEAR/2 (screen* or detect* or diagnos* or algorithm* or identif* or grading or graded or method*)
#16	MeSH descriptor: [Image Interpretation, Computer-Assisted] this term only
#17	computer NEAR/2 aid* NEAR/2 diagnos*
#18	EfficientNet NEXT B3
#19	AlexNet or DenseNet or GoogLeNet or MobileNet or NASNet or ResNet or Ensemble or Inception-v3 VGG16 or Xception
#20	Neural NEAR/1 Network NEAR/1 Classifier*
#21	segmentation NEAR/2 (imag* or network*)
#22	capsule NEAR/1 network*
#23	multi-label NEAR/1 classificat*
#24	Multiple NEAR/1 Lesion* NEAR/1 Insert*
#25	Multiple NEAR/1 Instance NEAR/1 Learn*
#26	Bagging
#27	Naive NEAR/1 Bayes
#28	Multilayer NEAR/1 Perceptron
#29	(multi-layer NEAR/1 perceptron) or MLP
#30	Radial NEAR/1 Basis NEAR/1 Function

#31	Random NEAR/1 Forest
#32	(Ada or gradient) NEAR/1 boost*
#33	LASSO
#34	Elastic NEAR/1 Net
#35	genetic NEAR/1 algorithm*
#36	(decision or classification or regression or probability or model*) NEAR/3 tree*
#37	(logistic* NEAR/2 regression NEAR/15 learn*)
#38	augment* NEAR/1 clinical NEAR/1 decision* NEAR/1 mak*
#39	nearest NEAR/1 (neighbor or neighbour)
#40	fuzzy NEAR/3 (logit or logic or logistic)
#41	kernel
#42	MeSH descriptor: [Datasets as Topic] this term only
#43	(Kaggle or EyePACS or Messidor or DIARETDB1 or DIARETDB0 or e-Ophtha or UoA-DR or IDRiD or Ophdiat or HEI-MED or DiaretDB or APTOS-2019)
#44	#1 or #2 or #3 or #4 or #5 or #6 or #7 or #8 or #9 or #10 or #11 or #12 or #13 or #14 or #15 or #16 or #17 or #18 or #19 or #20 or #21 or #22 or #23 or #24 or #25 or #26 or #27 or #28 or #29 or #30 or #31 or #32 or #33 or #34 or #35 or #36 or #37 or #38 or #39 or #40 or #41 or #42 or #43
#45	MeSH descriptor: [Diabetic Retinopathy] this term only
#46	(diabet* or proliferative or non-proliferative) NEAR/4 retinopath*
#47	diabet* NEAR/3 (eye* or vision or visual* or sight*)
#48	retinopath* NEAR/3 (eye* or vision or visual* or sight*)
#49	DR NEAR/3 (eye* or vision or visual* or sight*)
#50	#45 or #46 or #47 or #48 or #49
#51	#44 and #50
#52	(developing or less* developed or under developed or underdeveloped or middle income or low* income) near/2 (economy or economies):ti,ab,kw
#53	(developing or less* developed or under developed or underdeveloped or middle income or low* income or underserved or under served or deprived or poor*) near/2 (countr* or nation* or population* or world):ti,ab,kw
#54	low* near/2 (gdp or gnp or gross domestic or gross national):ti,ab,kw
#55	low near/3 middle adj3 countr*:ti,ab,kw
#56	lmic or lmics or third world or lami countr*:ti,ab,kw

#57 transitional countr*:ti,ab,kw

#58 MeSH descriptor: [Developing Countries] explode all trees

#59 MeSH descriptor: [Global Health] explode all trees

#60 MeSH descriptor: [Africa] explode all trees

#61 MeSH descriptor: [Caribbean Region] this term only

#62 MeSH descriptor: [Central America] this term only

#63 MeSH descriptor: [Latin America] this term only

#64 MeSH descriptor: [South America] this term only

#65 MeSH descriptor: [Asia] explode all trees

#66 MeSH descriptor: [China] explode all trees

#67 MeSH descriptor: [Pacific Islands] explode all trees

#68 #52 or #53 or #54 or #55 or #56 or #57 or #58 or #59 or #60 or #61 or #62 or #63 or #64 or #65 or #66 or #67

#69 #51 and #68
