# Supplementary material for: Artificial intelligence for diabetic retinopathy in low-income and middle-income countries: a scoping review
Source: BMJ Open Diabetes Res Care. 2023 Aug 2;11(4):e003424. doi: 10.1136/bmjdrc-2023-003424 (PMC10401245; doi:10.1136/bmjdrc-2023-003424)
Supplement: Supplementary data [file bmjdrc-2023-003424supp002.pdf]

# Publicly available datasets for diabetic retinopathy from low- and middle income countries

| | |
|---|---|
| Asia Pacific Teleophthalmology society | India |
| Retinal Fundus and OCT | Iran |
| DR1 | Brazil |
| DR2 | Brazil |
| Diabetic Retinopathy Image Database | Turkey |
| Fundus fluorescein angiogram and colour fundus | Iran |
| Fundus images with exudates | Iran |
| Indian Diabetic Retinopathy Image Dataset | India |
| Ocular disease intelligent recognition | China |
| Canada OCT retinal images | India |
| RetinaCheck- Microaneurysm | Netherlands and China |
| RetinaCheck- Scanning Laser ophthalmoscopy Vessel patch | Netherlands and China |
| RetinaCheck- Scanning Laser ophthalmoscopy Microaneurysm | Netherlands and China |
| Retinal Optical Coherence Tomography classification challenge | Iran |
