# Supplementary material for: Artificial intelligence for diabetic retinopathy in low-income and middle-income countries: a scoping review
Source: BMJ Open Diabetes Res Care. 2023 Aug 2;11(4):e003424. doi: 10.1136/bmjdrc-2023-003424 (PMC10401245; doi:10.1136/bmjdrc-2023-003424)
Supplement: Supplementary data [file bmjdrc-2023-003424supp003.pdf]

Supplementary table 1: *Details of the arbitration process for the externally validated studies included in Table 1 of the main manuscript*

Study	Details of arbitration process
Sosale et al [70]	5 persons graded each image with the majority grade considered as the gold standard
Bahl et al [71]	NS
Natarajan et al [72]	2 ophthalmologists graded each image. In case of discrepancy, a VR surgeon made the final decision
Astha et al[51]	Any disagreements were arbitrated by a third senior vitreo-retinal surgeon
Ting et al [67]	NS
Bellemo et al [53]	NS
Ting et al [27]	Varied between datasets
Sayres et al [73]	3 retinal specialists participated in multiple rounds of adjudication until consensus was reached
Raumviboonsuk et al [66]	When disagreements occurred, graders were able to re-grade images with access to their prior grade and other specialists grade. If agreement was still not reached over 3 rounds of this approach, a senior retina specialist assigned the final grade
Gulshan et al [74]	At Aravind all images were adjudicated by a panel of 3 senior retinal specialists who graded images independently with any disagreements discussed until consensus was reached.<br>At Sankara, if all graders agreed on final grade, that was accepted as the ground truth, otherwise the same protocol as at Aravind was used.
Gulshan et al [75]	An image was classified as referable if ≥50% of ophthalmologists graded it referable
Bora et al [40]	NS
Ruamviboosnuk et al[55]	Any disagreements were reviewed by adjudicators in a round-robin fashion until all panel members agreed.
Al-Turk et al [76]	NS
Al-Turk et al [77]	NS
Rajalakshmi et al [78]	Disagreements adjudicated by a third retinal specialist
Zhang et al [56]	Disagreements were reviewed by a senior grader and their decision was taken as final grade
Li et al [57]	N/A (only 1 grader)
Lin et al [25]	Disagreements resolved by an expert retinal specialist
Ming et al [79]	Disagreements were re-graded by a specialist panel
Pei Xiao Ting et al[80]	Disagreements were adjudicated by a third retinal specialist
Hao et al[58]	Disagreements were reviewed by a more senior chief physician who decided the final grade
Bawankar et al [81]	NS
Hansen et al [82]	NS
Dong et al[26]	Disagreements were reviewed by a third expert who made the final decision
Yang et al[59]	Disagreements were decided by a third senior grader
Nunez do Rio et al[52]	A senior ophthalmologist arbitrated disagreements
Mathenge et al[18]	NS
Li et al [68]	Disagreements adjudicated by a third senior grader
